# Hair testing for investigating intake and use history of hypnotics in the forensic field

**DOI:** 10.1007/s11419-025-00730-7

**Published:** 2025-07-07

**Authors:** Noriaki Shima, Munehiro Katagi, Takako Sato

**Affiliations:** 1 Forensic Science Laboratory, Osaka Prefectural Police Headquarters, 1-3-18 Hommachi, Chuo-ku, Osaka, 541-0053 Japan; 2 Division of Preventive and Social Medicine, Department of Legal Medicine, Osaka Medical and Pharmaceutical University, 2-7 Daigaku-machi, Takatsuki, Osaka 569-8686 Japan

**Keywords:** Hair testing, Drug use history, Hypnotics, Hair dyeing, Segmental analysis, LC–MS/MS

## Abstract

**Purpose:**

Hair testing for drugs has been used extensively in the forensic field since the 1990s, primarily in cases involving abused drugs such as methamphetamine and cocaine. Since the 2010s, its scope has expanded to include the detection of single dose of hypnotics, aiding in the investigation of serious crimes. This review presents essential knowledge for hair testing and the currently recommended analytical procedures and forensic applications.

**Methods:**

A review of literature from the 1990s to the 2020s was conducted, focusing on analytical methods for detecting drugs in hair, drug concentrations in hair, and drug incorporation pathways.

**Results:**

The characteristics of hair as a biological specimen include a longer detection window than other matrices such as urine and blood, as ingested drugs remain stable in hair over time. Significant differences in drug concentrations in hair are observed among substances, with several hypnotics, such as triazolam, having extremely low concentrations. Drugs are incorporated into hair primarily through two main pathways (the hair bulb and the upper dermis zone), with the dominant pathway depending on the drug’s properties. In addition, hair dyeing and subsequent exposure to aqueous environments (e.g., daily hair washing) can significantly influence drug concentrations and their distribution patterns (concentration and hair region). These factors must be carefully considered in hair testing.

**Conclusions:**

Hair testing is an effective means for proving drug intake and estimating use history, particularly in cases where there is a delay in reporting the incident. The interpretation of results must account for various factors, such as the chemical structures of drugs, incorporation pathways, and hair dyeing.

## Introduction

Hair, along with urine and blood, is one of the most widely used biological specimens in forensic investigations. In particular, hair testing for hypnotics has been helpful in verifying the ingestion of drugs that are exploited in serious crimes such as murder and drug-facilitated sexual assaults (DFSAs).

The first detection of hypnotics in hair was reported in 1992 when Sramek et al. [1] identified diazepam, a benzodiazepine, using radioimmunoassay. Subsequently, from 1996 to 2000, several research groups reported the detection of benzodiazepines (diazepam, nordiazepam, oxazepam, nordazepam, flunitrazepam (FNZ), clonazepam (CLO), and their metabolites) using gas chromatography-mass spectrometry (GC–MS) [2–5]. Thereafter, in 2000 and 2001, Negrusz et al. [5, 6] successfully detected 7-aminoclonazepam, FNZ, and 7-aminoflunitrazepam in hair from subjects who had taken a single dose of hypnotics (FNZ and CLO) using gas chromatography-negative chemical ionization-mass spectrometry (GC-NCI-MS). This work was of great significance, as single-dose ingestion is often assumed in murder and DFSA cases. However, this method required a large quantity of hair – 50 mg of 2-cm hair segments (approximately 250–500 strands) – making it difficult to apply to other low-dose hypnotics such as triazolam (TZ).

In the 2000s, liquid chromatography-mass spectrometry (LC–MS) emerged as a highly sensitive analytical method for detecting hypnotics, replacing GC-NCI-MS. By the late 2000s, the introduction of electrospray ionization (ESI), which can ionize a wide range of drugs, enabled liquid chromatography-tandem mass spectrometry (LC–MS/MS) to become the leading analytical method, allowing the detection of hypnotics in hair from subjects taking a single dose [7–12]. After 2015, as instrument sensitivity improved, drugs with higher concentrations in hair, such as zolpidem (ZP), could be detected using a single strand; this advancement facilitated research on drug incorporation pathways and time-course changes in drug distribution in hair [10, 13–15].

This review presents hair testing for the investigation of drug intake and use history, mainly focusing on benzodiazepines, non-benzodiazepines, and orexin receptor antagonists, which are the most commonly prescribed hypnotics in Japan. It also discusses future perspectives in the forensic field.

## Fundamental data for hair testing

### Drug detection

To determine whether target drugs can be detected in hair, it is essential to consider both the concentration levels of different drugs incorporated into hair and the sensitivity of the analytical instrument used. This is especially critical when testing for hypnotics in cases of murder and DFSA, where concentration data from a single intake is indispensable.

#### Drug concentration in hair

Detecting drugs in hair can be challenging due to their low concentration levels, especially with hypnotics. Table 1 shows the concentrations of hypnotics (pg/mg) in hair following a single intake. These concentration ranges (0.5–590 pg/mg) are significantly lower than the reported average concentrations of methamphetamine and cocaine in hair from chronic users (methamphetamine: 18,300 pg/mg (n = 15) [16]; cocaine: 12,900 pg/mg (n = 67) [17]). Among hypnotics, a significant concentration difference (54- to 1100-fold) was also observed when comparing estazolam (a benzodiazepine, black hair, 0.5–2.6 pg/mg [12]) and ZP (a non-benzodiazepine, black hair, 140–550 pg/mg [10, 11]), suggesting that the difficulty of detection depends on the specific hypnotic in question.

**Table 1:** Literature data on the concentrations of hypnotics and their metabolites in hair

Drug or metabolite	Dose (mg)	Number of subjects (black-haired subjects)	Concentration in hair (pg/mg)
Clonazepam [6]	2	2 (no information)	Not detected
7-Aminoclonazepam			4.8, < 1.0 (LOQ)
Flunitrazepam [7]	2	10 (2)	< 2.5 (LOQ)
7-Aminoflunitrazepam			0.5–8.0
Bromazepam [8]	6	2 (no information)	3.5, 28
Zolpidem [9]	10 (tartrate)	3 (no information)	1.8–9.8
Zolpidem [10]	10 (tartrate)	20 (20)	140–550
Zolpidem [11]	10 (tartrate)	1 [15 hair strands] (1)	140–340
Estazolam [12]	1	14 (14)	0.5–2.6
Zopiclone [13]	5 or 10	16 (0)	5.0–370 (5 mg), 17–590 (10 mg)
N-Desmethylzopiclone			5.4–300 (5 mg), 25–410 (10 mg)

#### Concentration unit

Drug concentration in hair in most studies are expressed as “drug amount per weight of hair (pg/mg),” as shown in Table 1. However, using this unit makes it difficult to estimate the number of hair strands required for drug detection unless information for unit conversion (hair weight per length) is provided. In addition, extreme caution is needed when comparing drug concentrations under conditions of single intake. Since the drug may be localized in a specific segment of the hair strand, the measured drug concentration will vary depending on the size of the analyzed hair fraction. Therefore, when comparing concentrations, the fraction size must be taken into account. To improve these problems, the author's group has proposed using the absolute amounts of drugs in a single hair strand (pg/hair strand) as a standardized unit for quantification [10, 13, 18]. This approach facilitates data comparison across research groups and allows for the estimation of the number of hair strands needed for hair testing.

The data presented in Table 1 are directly comparable, as they were all obtained using 2-cm hair segments.

#### Number of hair strands for the testing

Table 2 shows the amounts of seven drugs in hair (four benzodiazepines, one non-benzodiazepine ZP, and two orexin receptor antagonists) as reported by Sasaki et al. and Nitta et al. in 2021 and 2025, respectively [18, 19]. These data were obtained from subjects (with black hair) who had taken a single dose of drugs, approximately one month before hair collection. Drug quantification was performed using the “pg/hair strand” as the unit (Table 2A), and the values were also converted to the conventional “pg/mg” unit (Table 2B) based on the average hair weight (mg/hair strand). This conversion allows for direct comparison with previously reported data (Table 1).

**Table 2:** Amounts of hypnotics and metabolites detected in hair approximately one month after intake, along with the estimated incorporation ratios (%), representing the rough percentage of the administered dose incorporated into head hair

	Drug or metabolite	Dose (mg)	Number of subjects	(A)	(B)	(C)
				Detected amount (pg/hair strand)	Drug concentration in hair (pg/mg)	Ratio (%) of total amount of the drug incorporated into scalp hairs to dose (supposing to be 100,000 strands)
					Calculation (A)/hair weight (2-cm segment)	Calculation ((A) × 100,000/dose) × 100
Benzodiazepines [18]	Triazolam	0.25	3	0.036	0.32	0.0014
	Etizolam	1	3	0.35	3.0	0.0035
	Flunitrazepam	2	3	1.1	9.2	0.0055
	7-Aminoflunitrazepam			7.4	66	0.041
	Nitrazepam	5	2	1.6	15	0.0032
	7-Aminonitrazepam			4.4	41	0.010
Non-benzodiazepines [18]	Zolpidem	10 (tartrate)	3	25	220	0.031
Orexin receptor antagonists [19]	Suvorexant	10	3	0.036	0.18	0.000036
	Lemborexant	5	3	0.13	0.70	0.00026

Among the drugs in Tables 1 and 2, the lowest concentrations were observed for TZ (0.25 mg/tablet) and suvorexant (SUV, 5 mg/tablet), both averaging 0.036 pg/hair strand (0.32 pg/mg and 0.18 pg/mg, respectively). In real forensic cases, the specific hypnotic ingested is often unknown, and therefore the number of hair strands required for drug testing should be determined based on the hypnotic that is incorporated into hair at the lowest concentration. For instance, if the instrument (LC–MS/MS) requires 1.0 pg of a drug to identify a compound, approximately 30 hair strands would be needed to detect TZ and SUV (1.08 pg/30 hair strands). On the other hand, ZP, which has the highest concentration in hair (25 pg/hair strand), can be detected using a single strand. However, if the instrument’s sensitivity were reduced to 1/100 of its original performance, 3,000 strands would be required to detect TZ and SUV. In such a case, hair testing could be declined due to the excessive burden imposed on the victim.

Regarding drug metabolites, Tables 1 and 2 show that the 7-amino metabolites of nitro-containing benzodiazepines (FNZ, CLO, nitrazepam (NZ)) were detected at higher concentrations than their parent compounds. However, hydroxyl metabolites of TZ and other hypnotics, as well as carboxyl metabolites of ZP, have not been reported in hair. This suggests that highly polar metabolites are poorly incorporated into hair. Thus, for benzodiazepines with hydroxyl or carboxyl groups in their structures (e.g., chlorazepic acid, flutazolam, lorazepam, and lormetazepam), the hair concentrations could be lower than those for TZ and SUV. Further studies are needed to determine whether these drugs can be reliably detected in hair. Notably, one case report from 2004 documented the absence of lorazepam in authentic hair specimens [20].

#### Distribution ratio of drugs to head hair tissue

The ratio of a drug distributed in head hair tissue to the amount ingested can be roughly estimated based on the drug concentration in a single strand (Table 2A). Sasaki et al. and Nitta et al. calculated the distribution ratios of hypnotics in hair tissue (Table 2C), assuming an average number of human head hairs is 100,000. The ratios for seven hypnotics ranged from 0.000036% to 0.031%, which is significantly lower than the urinary excretion ratios (cumulative urinary excretion ratios) of the respective hypnotics including their metabolites [21–31].

The distribution ratios of four benzodiazepines (TZ, etizolam, FNZ, and NZ) ranged from 0.0014 to 0.0055% (Table 2C), which are relatively similar. This suggests that the amounts of benzodiazepines incorporated into hair are roughly proportional to the administered dose. Therefore, the distribution amounts of other benzodiazepines, for which hair incorporation data are unavailable, could be estimated based on their doses. However, as mentioned above, for benzodiazepines containing hydroxyl or carboxyl groups, the distribution ratios could be significantly lower, making estimations difficult without specific data.

Meanwhile, the distribution ratio of ZP (a non-benzodiazepine), was 0.031%, approximately 10 times higher than that of benzodiazepines. ZP’s high lipophilicity and basicity likely contribute to this easier incorporation into hair. As previously reported by Nakahara et al. [32, 33], highly lipophilic and basic compounds tend to be readily incorporated into hair. However, two orexin receptor antagonists (SUV and lemborexant) exhibited very low distribution ratios (0.000036% and 0.00026%, respectively) despite their high lipophilicity. This may be due to their strong affinity for melanin pigments, preventing extraction from hair and resulting in deceptively low distribution ratios. The causes of the low distribution ratios require further studies in the future.

Since determining the actual amounts incorporated into hair for all drugs is challenging, the authors suggest that estimating drug incorporation based on chemical structure and dosage is also important.

#### Influence of hair coloration

Drug incorporation into hair is mainly influenced by two factors. One is the drug’s chemical structure (lipophilicity and basicity) as discussed in the previous section, and another is the pigment component (melanin pigment) in hair. There are two main types of melanin pigments: eumelanin (black or dark brown) and pheomelanin (yellowish to reddish colors), of which eumelanin is considered to be strongly involved in drug incorporation [34]. Eumelanin is believed to have a strong affinity for lipophilic and basic compounds due to its chemical structure: studies have shown that basic drugs of abuse, such as methamphetamine, cocaine, and codeine, are rapidly incorporated into black hair with high eumelanin content [32–37], and their hair concentrations correlate with melanin levels [36, 37]. On the other hand, neutral and acidic compounds with low melanin affinity (e.g., N-acetylamphetamine [38] and ethanol metabolites such as ethyl glucuronide [39] and fatty acid ethyl esters [40]) show no significant concentration differences between black and white hair.

Regarding hypnotics, Miyaguchi et al. studied two non-benzodiazepines (ZP [41] and zopiclone [42]) in individuals with gray hair who ingested daily doses (ZP: 10 mg tablet/day, zopiclone: 5 mg tablet/day). ZP was detected at concentrations of 18,300 pg/mg in black hair and 119 pg/mg in white hair, while zopiclone was detected at > 10 ng/mg in black hair and 187 pg/mg in white hair. Both drugs are lipophilic and basic with high affinity for melanin pigments, leading to remarkable concentration differences between black and white hair. For ZP, Villian et al. reported hair concentrations ranging from 1.8–9.8 pg/mg after a single dose [9], whereas Cui et al. and the authors reported much higher concentrations of 140–550 pg/mg [10] and 140–340 pg/mg [11], as shown in Table 1. These discrepancies likely stem from differences in pretreatment (drug extraction methods) [43]; however, since the color of the subjects' hair was not reported, variations in eumelanin content in hair could also be a contributing factor. In addition, the difference in ZP distribution between black and white hair was investigated in detail using segmental hair analysis by the authors [14], which will be discussed later.

For FNZ, Negrusz et al. [6] reported that in 10 subjects (2 of whom with black hair), FNZ was detected at concentrations below the limit of quantification (LOQ: 2.5 pg/mg hair), while its metabolite 7-aminoflunitrazepam was detected at concentrations ranging from 0.5 to 8.0 pg/mg as shown in Table 1. On the other hand, Sasaki et al. [18] found that FNZ and 7-aminoflunitrazepam were detected at concentrations of 9.2 pg/mg and 66 pg/mg, respectively, in three subjects (all with black hair) (Table 2B). These differences in concentration between studies may be explained by variations in hair color.

Thus, even when hypnotics are ingested under identical conditions, drug concentrations in hair can vary significantly based on hair color. This indicates that the number of hair strands to be collected in forensic cases should be determined with hair pigmentation in mind.

### Estimation of drug use history

Hair testing can provide not only proof of drug intake, but also an estimate of drug use history (the date and amount of intake), often serving as important evidence to prove a crime. The use history is calculated based on the exact positions and amounts of drugs distributed in hair. Analysts must rigorously understand the pathways by which drugs incorporate into hair and the time-course changes in their distribution. This section presents findings on drug incorporation and distribution, as well as hair structure.

#### Structure of head hair and the hair cycle

**Fig. 1:**
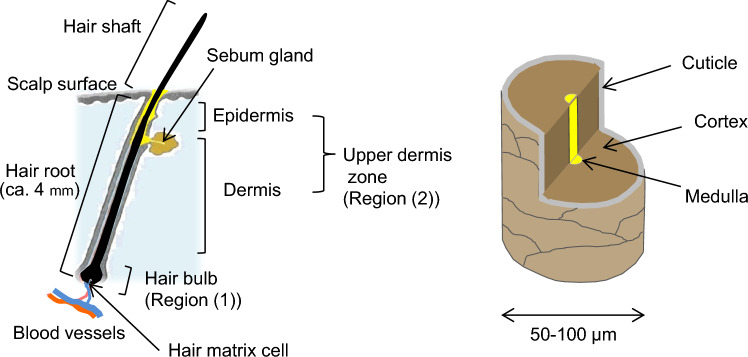
Schematic diagram of a head hair

Figure 1 illustrates the structure of head hair, and Table 3 summarizes the hair cycle and average growth rates of head and body hair [44, 45]. Hair is produced by the division of hair matrix cells in the bulb, and is pushed toward the scalp as it keratinizes, growing at an average rate of 0.3–0.5 mm/day (0.9–1.5 cm/month) during the growth (anagen) phase. The anagen phase lasts for 2 to 6 years, followed by the regressing (catagen) phase of about 2 weeks, the resting (telogen) phase lasting 2 to 6 months, and the exogen phase, during which old hair is replaced by new hair and sheds. Since ingested drugs are incorporated into growing hair, it is theoretically possible to detect them in hair for 2 to 6 years after intake. Approximately 6–18% of hair strands are in the catagen and telogen phases at any given time (Table 3). During these phases, hair growth slows or stops, and drug incorporation is minimal, resulting in individual differences in the positions and amounts of drugs distributed in hair. Thus, the hair cycle is a critical factor in hair testing.

**Table 3:** Literature data on hair cycle duration, the proportion of hair in the catagen + telogen stages, and growth rate [44, 45]

Anatomical site	Duration of stages		Portion in (catagen + telogen) stage	Growth rate
	Anagen	Catagen + Telogen	%	mm/day
Head	2–6 years	2–6 months	6–18	0.3–0.5
Beard	14–22 months	9–12 months	40	0.3
Axillary	11–18 months	12–17 months	50	0.3
Pubis	11–18 months	12–17 months	50	0.3

Head hair is about 50–100 μm thick and consists of three-layers: the cuticle, cortex, and medulla (Fig. 1). The cortex, which accounts for 90% of hair’s mass, contains macrofibrils (fiber bundles) and melanin pigments that determine hair color. It is believed that incorporated drugs are primarily located with these pigments in the cortex. Depending on the cuticle’s condition, drugs in the cortex may be leached or degraded by shampoo or UV light, factors that must be taken into consideration when testing damaged hair.

#### Drug incorporation pathways into hair

Understanding the pathways through which drugs incorporate into hair is essential for estimating drug use history, as these pathways influence drug distribution (position and amount). Extensive studies on drug incorporation were conducted in the 1990s [45–48]. According to these reports, drugs absorbed into the body are transported along with nutrients in the bloodstream to the hair bulb, where they bind to hair tissue or melanin pigments during keratinization, and subsequently migrate to the hair shaft (the exposed area outside the scalp) as hair grows (Fig. 1). However, several studies have indicated that the incorporation pathway is not limited to the hair bulb located at the root’s base; alternative pathways exist through the upper part of the hair root via sebum and sweat [46–50]. Studies from the 1990s to approximately 2010, on which the above theory is based, investigated drug distribution using segmental analysis of 0.5 to 2 cm hair sections from multiple strands. However, due to individual differences in hair growth rate and hair cycle stages (anagen, catagen, and telogen), this approach had limitations in accurately determining drug distribution shape (height and width) and refining incorporation pathway models.

Although the drug incorporation mechanism has not been thoroughly elucidated, recent advances in analytical instruments and techniques have gradually clarified the behavior of drugs in hair. The clarification was attributed to the detailed observation of drug distribution in a single strand. Since 2015, ZP has been selected as a model drug, and its distribution has been studied in detail to provide new insights into incorporation pathways [13, 14].

##### Distribution of drug in hair 1 and 2 months after intake

**Fig. 2:**
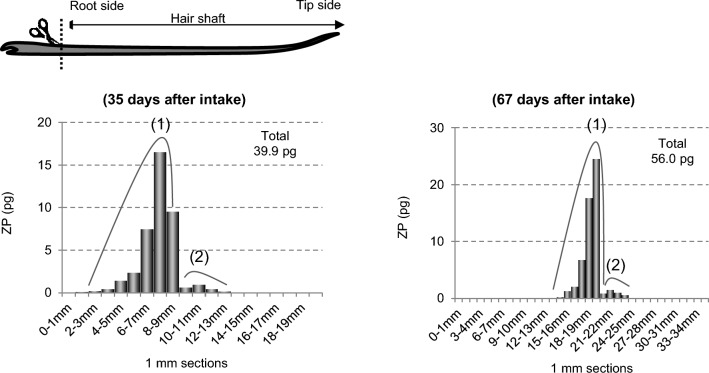
Amount of ZP in each 1-mm segment along single hair strands collected by cutting from a subject at 35 and 67 days after a single oral intake of 10 mg of ZP tartrate [13]. *ZP* zolpidem

A typical example of the distribution of ZP in hair approximately 1 month (35 days) and 2 months (67 days) after intake, as reported by the authors in 2017, is shown in Fig. 2 [13]. Despite a single intake, ZP was distributed over a 10 mm width in both samples, corresponding to approximately 25 days of hair growth (growth rate: 0.4 mm/day). Given ZP’s biological half-life of 2 to 3 h, its blood concentration should become undetectable within 1 to 2 days after intake. However, ZP remained distributed over a width equivalent to approximately one month of hair growth.

Focusing on the concentration, two peaks (peaks (1) and (2)) were observed (Fig. 2). This pattern was reproducible across multiple subjects, with peak (1) near the root side showing maximum values of approximately 15–30 pg/1-mm hair, while peak (2) near the tip side showed approximately 1 pg/1-mm hair [13].

As described above, there were no significant differences in the distribution width and shape, or the drug amount in hair between one and two months after intake. This suggests that ZP in the hair shaft is resistant to degradation, spreading, or removal by external factors such as shampoo or UV light. The observed shift in ZP’s position, approximately 12 mm toward the tip over one month (67 days − 35 days = 32 days), corresponds to a migration rate of 0.38 mm/day, consistent with typical hair growth rates (0.3–0.5 mm/day). This finding supports the idea that ZP remains fixed within the hair and migrates toward the tip end as the hair grows, while maintaining its distribution shape.

Traditionally, drugs were believed to be incorporated into hair primarily through the hair bulb, which is located at the bottom of the hair root (about 4 mm) (Fig. 1). However, the observation that ZP was distributed over a 10 mm width in hair shafts even after a single intake, and retained its distribution shape over time, suggests a more complex incorporation process. To elucidate how this 10-mm distribution shape forms, further studies were conducted on hair samples collected in the early stage (10 h to 35 days) after intake.

##### Time course changes in drug distribution in hair from 10 h to 35 days after intake

**Fig. 3:**
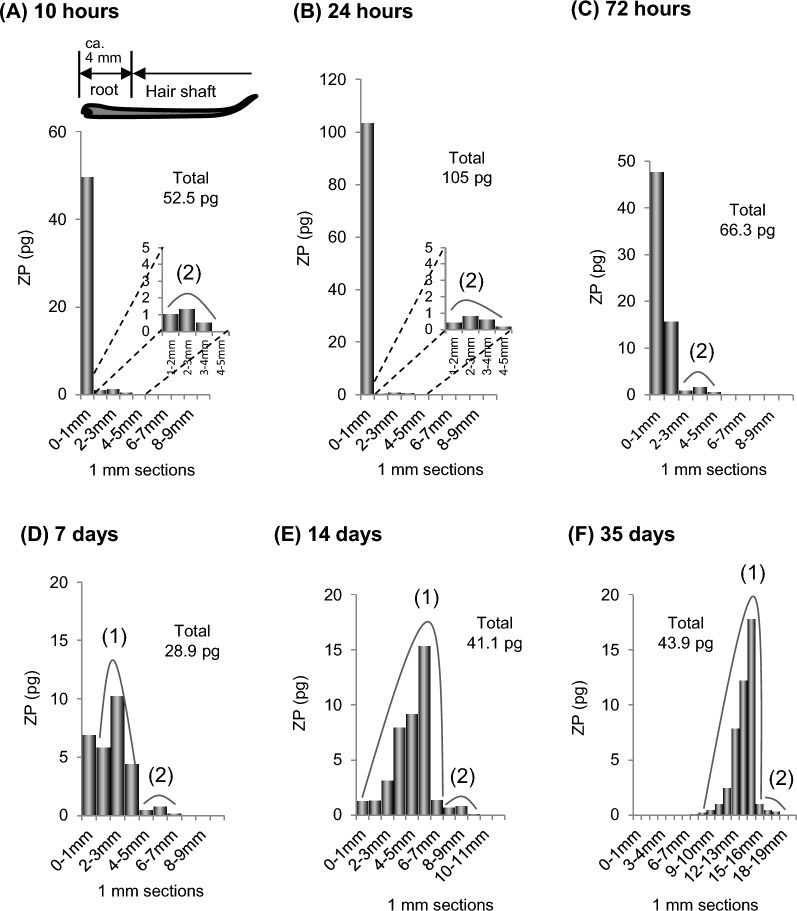
Amount of ZP in each 1-mm segment along single hair strands collected by plucking from a subject at interval ranging from 10 h to 35 days after a single oral intake of 10 mg of ZP tartrate [13]. *ZP* zolpidem

Figure 3 shows the time course changes in drug distribution from 10 h to 35 days after a single intake of ZP [13]. Hair samples were collected by plucking with the roots intact, and drug distribution was investigated mainly focusing on the hair root. ZP was detected as early as 10 and 24 h after intake, with its distribution extending throughout the hair root (4–5 mm). The highest concentration (50–100 pg/1-mm hair) was observed in the hair bulb (0–1 mm section from the root end), while lower concentrations (0.1–1.0 pg/1-mm hair) were observed in upper part of the hair root (1–5 mm section), where peak (2) had already formed, reaching a maximum value of about 1.0 pg/1-mm hair in the 2–3 mm segment. Thereafter, the distribution region of ZP continued to extend from 72 h to 14 days after intake, and by 35 days, ZP had shifted to the hair shaft (outside the scalp), extending to approximately 10 mm.

Despite a typical hair growth rate of 0.3–0.5 mm/day, ZP was detected throughout the hair root (4–5 mm wide) at 10 and 24 h after intake, with peak (2) already present in the upper root. This suggests that drug incorporation occurs via two pathways: the hair bulb (region (1)) and the upper dermis zone (region (2)). Drug incorporation through region (1) persisted for about two weeks, with the amount of incorporation gradually decreasing, leading to an extended distribution width and the formation of peak (1). The distribution shapes observed between 72 h and 14 days after intake, along with peak concentrations (10–20 pg/1-mm hair), suggest that only a portion (approximately 1/4 to 1/5) of the drug in region (1) was incorporated into keratinized hair and migrated toward the tip side.

On the other hand, drug incorporation through region (2) appeared to be complete within 10 to 24 h after intake, with a distribution width of 3–4 mm. At this stage, peak (2) had already formed and thereafter migrated toward the tip side while maintaining its shape up to 35 days after intake. Since the upper hair root is already partially keratinized, direct incorporation via the bloodstream is unlikely. Instead, it is suggested that drugs are incorporated into region (2) through infiltration of sebum secreted by adjacent sebaceous glands (Fig. 1).

In summary, detailed single-strand analysis has demonstrated that drug incorporation into hair routinely involves two distinct pathways.

##### Differences in distribution shape between drugs

**Fig. 4:**
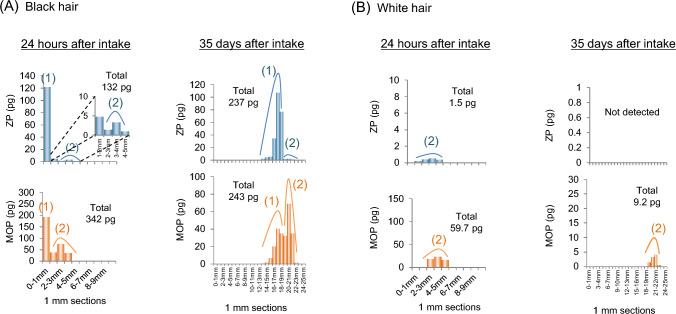
Amounts of ZP and MOP in each 1-mm segment along **A** black and **B** white single strands collected by plucking at 24 h and 35 days after ZP intake [14]. *ZP* zolpidem, *MOP* methoxyphenamine

Drugs are incorporated into hair through two pathways as described in the previous section. However, the distribution shapes vary depending on the characteristics of drugs. As shown in Fig. 2, the amount of ZP incorporated through region (1) is greater than that through region (2), resulting in a distribution shape where peak (1) is higher than peak (2). On the other hand, certain compounds, such as ethyl glucuronide, a highly polar neutral metabolite of ethanol, reported by Schräder et al. [50], are not incorporated through region (1) but mainly through region (2). Similarly, the distribution shape of methoxyphenamine (MOP, a model drug for methamphetamine) in hair was reported by the authors [14] and Nitta et al. [51], which showed that MOP is incorporated through both regions (1) and (2) at similar levels, leading to comparable heights for peaks (1) and (2) as shown in Fig. 4A.

As described above, the distribution shape of a drug or metabolite depends on its primary incorporation pathway, which, in turn, is influenced by the compound’s characteristics (lipophilicity and basicity). In other words, highly lipophilic basic compounds, such as ZP, are primarily incorporated through region (1), whereas highly polar neutral compounds, such as ethyl glucuronide, are incorporated mainly through region (2) due to their poor incorporation through region (1). Incorporation through region (2) likely occurs due to sebum infiltration into the upper part of the hair root, suggesting that even highly polar compounds can be incorporated into hair when contained in secreted sebum.

Thus, understanding these differences in distribution shapes is important when estimating drug use history, and accumulating data on distribution shapes for various drugs will be essential for future analysis. The predominant incorporation pathway of a target drug can be estimated from the degree of distribution ratio (high or low) to the hair tissue. Drugs with a high distribution ratio, such as ZP, are predicted to be mainly incorporated through region (1), while drugs with a low distribution ratio, such as alcohol metabolites, are more likely to be incorporated through region (2). Benzodiazepines, often used in murder and DFSA cases, have lower distribution ratios than ZP, suggesting that, like MOP, they are incorporated through both regions (1) and (2) at similar levels. This trend has been observed in actual forensic cases.

##### Influence of melanin pigment (distribution of drugs in white hair)

Melanin pigment is closely involved in drug incorporation, with notable differences in the amounts of highly lipophilic and basic drugs detected in black versus white hair, as described above. However, even in white hair, which lacks melanin pigment, drugs are not completely undetectable, but are present at low levels. The authors examined the distribution shapes of ZP and MOP in white hair in detail, highlighting the difference from black hair and discussing the role of melanin pigment [14]. Figure 4B shows the distribution shapes of drugs in white hair at 24 h and 35 days after ZP and MOP intake. At 24 h after intake, both ZP and MOP were incorporated through the region (2), with no detectable incorporation through region (1). This indicates that melanin pigment is necessary for incorporation through region (1), whereas melanin pigment is not involved in drug incorporation through region (2). Given that region (2) incorporation is likely caused by sebum infiltration, this further supports the idea that melanin pigment is not involved in this incorporation process.

At 35 days after intake (Fig. 4B), ZP was no longer detected in white hair, and the concentration of MOP was also significantly lower than at 24 h after intake. In contrast, in black hair (Fig. 4A), there was no significant change in drug levels over time, suggesting that melanin pigment plays a role in retaining drugs in hair after incorporation. Thus, melanin pigment appears to serve two roles: facilitating drug incorporation through region (1) and retaining drugs in hair once they are incorporated. Although the distribution shapes of drugs in white hair differ completely from those in black hair, white hair can still be used for drug testing by properly understanding its characteristics. However, it is necessary to consider the possibility that drug concentrations in hair may decrease over time.

## Overview of hair testing

Hair testing is primarily performed to prove drug intake and, if possible, to estimate the date and amount of intake. Based on the findings presented in the previous sections, this section provides recommendations for appropriate procedures ranging from requirements for hair testing to analytical methods.

### Requirements for hair testing

Before conducting hair testing in forensic investigations, it is first necessary to determine whether hair testing is worthwhile. At least two conditions must be considered: (1) hair length and (2) whether the hair has been bleached or dyed.

#### Hair length

Hair specimens should be sufficiently long to verify drug ingestion at the victimization date. This can be calculated based on the average growth rate of head hair (0.3–0.5 mm/day). For example, if the incident occurred one year ago, the approximate position of the drug in hair can be calculated as “0.3–0.5 mm/day × 365 days = 109.5–182.5 mm”, and thus in this case, hair testing would be worthwhile if the hair specimen is approximately 20 cm or longer.

#### Bleaching and dyeing

When hair is bleached or dyed, drugs can be degraded or leached out of hair due to the degradation of melanin pigment [52]. Hair dyeing includes three typical types: (1) permanent coloring, (2) hair manicure (semi-permanent coloring), and (3) hair mascara (temporary coloring). Of these, (1) permanent coloring involves a bleaching process, and therefore drugs may no longer be detectable if the subject’s hair was dyed after the incident. Thus, reviewing the history of bleaching and dyeing (including the dates and frequency) is necessary before determining whether hair drug testing should be performed.

If hair testing cannot be performed using head hair due to bleaching, dyeing, or lack of head hair, alternative hair specimens (e.g., pubic hair) may be used. However, as shown in Table 3, other hairs, such as pubic and axillary hairs, have different growth rates and hair cycles from head hair, making the interpretation of how drugs are incorporated into hair more complex. Therefore, while testing these hair types is possible [53], the results should be interpreted with caution.

### Hair collection

#### Procedure

Once the decision has been made to perform hair testing, hair specimens are collected from the subject. The specimens are collected from the posterior parietal region, which contains the largest number of hairs at the anagen stage. The hairs should be cut as close to the scalp as possible using scissors. The required number of hair strands depends on the detection sensitivity of the analytical instruments used in the laboratory and the amount of the target drug in the hair strand (Table 2A). In addition, the orientation of the hair strands (the root and tip) must be clearly identified for estimating the drug use history.

#### Date of hair collection

Drugs are incorporated into hair through two primary pathways: the hair bulb (region (1)) and the upper dermis zone (region (2)) as shown in Fig. 1. Since drug incorporation through region (1) has been demonstrated to continue for about 2 weeks after intake [13], it takes approximately 3–4 weeks, based on the average hair growth rate, for the drugs to be fully exposed outside the scalp. As described in the previous section, because hair specimens are collected by cutting them close to the scalp, it is recommended that the specimens be collected at least 4 weeks (approximately one month) after the incident. In other words, if less than one month has passed since the incident, it is advisable to wait until a full month has passed before collecting the specimen. If more than one month has passed, specimens should be collected as soon as possible.

### Qualitative analysis

#### Extraction of drugs from hair

Several methods for extracting drugs from hair have been investigated and reported. Typical methods include: (1) immersion extraction with methanol [54], (2) digestion of hair with sodium hydroxide [55], (3) immersion extraction with aqueous acids or buffer solutions [56], (4) supercritical fluid extraction [57], and (5) micropulverization extraction [41, 58]. Among these, (5) micropulverization extraction is considered the most efficient and rapid method for extracting various drugs. When extracting hypnotics (benzodiazepines and non-benzodiazepines) from hair using a stainless-steel bullet (crusher) and extraction solvent in a disposable 2 mL polypropylene tube, it is recommended that the amount of hair samples be less than 5 mg and that a pH 8.4 buffer solution be selected as the solvent [14, 18]. After drug extraction, the extracts are purified by liquid–liquid extraction or solid-phase extraction, and then concentrated and submitted to the instrument (LC–MS/MS).

#### Detection

A screening analysis is performed to detect targeted drugs and their metabolites that may be abused in serious crimes [59, 60]. In Japan, hypnotics include prescription drugs such as benzodiazepines (TZ, etc.), non-benzodiazepines (ZP, etc.), orexin receptor antagonists (SUV, etc.), melatonin receptor agonists (ramelteon), and over-the-counter drugs such as diphenhydramine, totaling approximately 30 to 40 drugs. When hypnotics are detected in hair, it is necessary to confirm that the victim was not self-medicating and that the suspect had access to the drug; this helps determine whether the drug was involved in the crime. However, special caution is required when diphenhydramine is detected. Diphenhydramine is also contained in antipruritic (anti-itch) ointments and may have been unknowingly used by the victim. As recently reported by Sasaki et al. [61], when applied topically as an ointment, diphenhydramine can be detected in hair as well as in blood and urine, and it is therefore necessary to carefully determine whether the drug was deliberately used in the context of a crime.

### Segmental analysis

By quantitatively investigating the position of the detected drug in hair, it is possible to estimate the drug use history, including the date and amount of intake. Two analytical methods are commonly used for this purpose: segmental analysis using LC–MS/MS, and mass spectrometry imaging (MSI) using matrix-assisted laser desorption/ionization mass spectrometry (MALDI-MS) [13, 62]. However, segmental analysis is typically preferred because MSI currently lacks the sensitivity to accurately quantify the low concentration levels of hypnotics. As mentioned above, the drug distribution shape should be identified using a single hair strand, and multiple strands (approximately 5 strands) should be analyzed to account for the ratio of hair strands at the catagen and telogen stages (6–18%) (Table 3). The fraction size of hair strand must also be carefully selected for segmental analysis. The smaller the fraction size, the more detailed the drug distribution can be, but the detectable amount of a drug decreases, leading to difficulty in detection and increases both preparation effort and analysis time. Thus, it is recommended that the fraction size (0.4 to 10 mm) be determined according to the above conditions and the needs of each case to prove a crime. When analyzing hypnotics with low concentrations in hair, such as TZ, multiple hair strands need to be used to ensure successful drug detection. It should be noted that the estimation accuracy may be lower in such cases.

## Applications to forensic cases

### Case 1

**Fig. 5:**
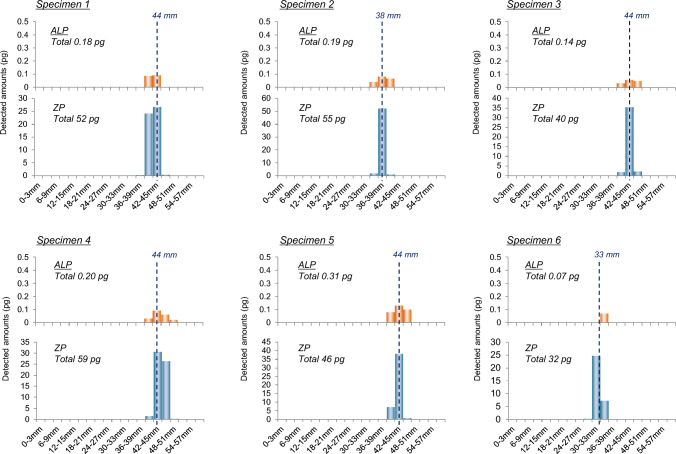
Amounts of ZP and ALP in each 3-mm segment along single strands (Specimens 1–6) collected from a victim in a DFSA case by cutting as close as possible to the scalp about 4 months after the incident [18]. *ZP* zolpidem, *ALP* alprazolam

Sasaki et al. presented a case report involving hair specimens (black) collected from a victim who had been sexually assaulted approximately 4 months earlier [18]. ZP and alprazolam (ALP, a benzodiazepine) were detected in the hair, and segmental analysis was performed on six individual hair strands (Fig. 5). In this case, a fraction size of 3 mm was selected because the concentration of ALP in hair was low. The results obtained from the segmental analysis objectively indicate the following evidence.

#### Date of intake

Both hypnotics were localized within 3 to 4 sections of the hair, indicating that the victim was not a frequent user of ZP and ALP. If the victim had been a frequent user, the drugs would have been detected over a wider range along the hair strand. The distribution width was 3 to 4 sections (approximately 10 mm), which is consistent with a single intake.

Two different methods have been reported for estimating the date of intake [15, 18]. One is based on the average growth rate of human head hair, as described in this section [18]. While this method introduces some error due to variations between the average and actual hair growth rates, it allows for relatively rapid processing from hair collection to testing. The other method, as reported by Kuwayama et al. [15], involves administering an indicator drug (e.g., chlorpheniramine) to the subject on two separate occasions after the incident, and the date of intake is estimated by determining the hair growth rate (measured value) from the distribution of the indicator drug. Although this method may not be suitable to DFSA cases due to the time required to collect hair samples (1–2 months) and ethical concerns, it is considered to have a smaller margin of error. The former method was used in this case.

As shown in Figs. 1 and 3, ZP is primarily incorporated through region (1), meaning that the position of the concentration peak of ZP corresponds to the location of the hair bulb when the drug was ingested. The formula for estimating the date of intake is as follows:

$$\begin{array}{*{20}l} {{\text{Days from drug ingestion to hair collection}} = } \hfill \\ {\quad \quad \left\{ {\left[ {{\text{position of ZP concentration peak }}\left( {{\text{distance from root side}},{\text{ mm}}} \right)} \right]*} \right.} \hfill \\ {\left. {\quad \quad + \, \left[ {{\text{4 mm }}\left( {\text{length of the hair root}} \right) \, + {\text{ 1 mm }}\left( {\text{thickness of scissors}} \right)} \right]*} \right\}} \hfill \\ {\quad \quad /\left[ {0.{\text{4 mm}}/{\text{day }}\left( {\text{average growth rate of head hair}} \right)} \right].} \hfill \\ \end{array}$$

*Since hair specimens were collected by cutting with scissors close to the scalp, it is assumed that the total length (5 mm) of the hair root (4 mm, Fig. 1) and the thickness of the scissors (1 mm) was lost during the cutting process.

If hair specimens are collected by plucking with the roots intact, this 5 mm should be removed from the formula.

Since the positions of concentration peaks of ZP, were on average, 41 mm (range: 33–44 mm, n = 6) from the root side, the result of “[41 mm] + [5 mm])/[0.4 mm/day] = 115 days” can be calculated by the above formula. The position “33 mm” for Specimen 6 may be considered an outlier, likely due to the hair being in the telogen stage. Even if this value is excluded, the calculated result would be 119.5 days, which remains consistent with the reported ingestion approximately 4 months prior. Two hypnotics (ZP and ALP) were detected in the same hair fraction for each specimen, indicating that the victim ingested both drugs at the same time (within 5 to 7 days of each other). The data does not contradict the possibility that the drugs were ingested on the same day.

As described above, the estimation of frequency and the date of intake by hair testing cannot provide definitive conclusions, but it can serve as valuable corroborative evidence in forensic investigations.

#### Amount of intake

The average amounts of ZP and ALP detected in individual hair strands were 47 pg/hair strand (range: 32–59 pg/hair strand) and 0.18 pg/hair strand (range: 0.07–0.31 pg/hair strand), respectively, as shown in Fig. 5. The amount of ZP ingested was estimated to be more than 1 tablet (10 mg tablet), based on quantitative data reported by Sasaki et al., which measured 36 pg/hair strand 3 months after intake (Table 4) [18]. On the other hand, since there is no previous report on ALP concentrations in hair, an estimate was made by referring to quantitative data for etizolam, another benzodiazepine with a similar dosage. Etizolam was detected at 0.14 pg/hair strand (Table 4), suggesting that the amount of ALP ingested was approximately 1 tablet (0.8 mg tablet, Solanax). These data indicate that the victim ingested sufficient amounts of the drugs to induce hypnotic effects.

**Table 4:** Amounts of five hypnotics detected in hair three months after intake [18]

Hypnotics	Dose (mg)	Average of detected amounts (pg/hair strand)
Triazolam	0.25	0.03 (n = 3)
Etizolam	1	0.14 (n = 3)
Flunitrazepam	2	0.38 (n = 3)
Nitrazepam	5	0.58 (n = 2)
Zolpidem	10 (tartrate)	36 (n = 3)

When estimating drug intake amounts, individual variations in drug incorporation into hair can introduce significant errors. However, these values are expected to be useful as references to determine whether the victim consumed a dose high enough to induce hypnotic effects.

### Case 2

**Fig. 6:**
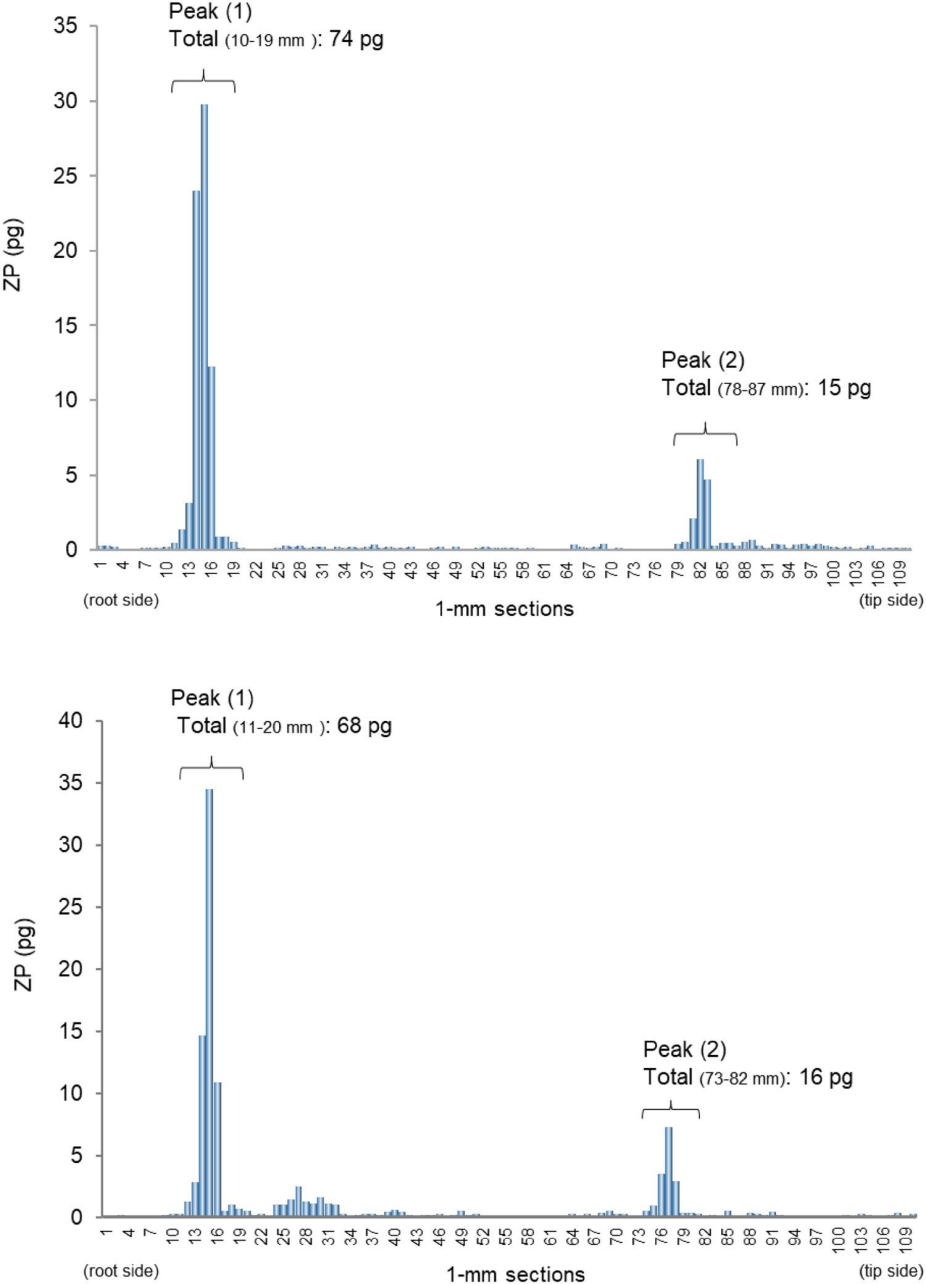
Amounts of ZP in each 1-mm segment along two single strands collected from a DFSA victim who was exposed to ZP twice, two and seven months before hair collection, and who received permanent hair coloring twice, two weeks and four months before hair collection. Hair specimens were collected by cutting using the scissors as close as possible to the scalp [63]. *ZP* zolpidem

The authors reported a case involving hair specimens (dyed brown) collected from a DFSA victim who had dyed their hair after the incident [63]. The victim was assaulted 2 and 7 months prior to hair collection, and had undergone permanent hair coloring twice, once 2 weeks prior and again 4 months prior to hair collection. ZP was detected in the specimens, and segmental analysis (1 mm sections) was performed for two individual strands (Fig. 6). The results of the segmental analysis objectively indicated the following evidence.

#### Date of intake

The distribution shape of ZP in the two hair strands were very similar, with two distinct concentration peaks (peaks (1) and (2)). The dates of drug intake were calculated from these peak positions using the formula described in the previous section. The results were consistent with the reported ingestion events occurring approximately 2 and 7 months prior, as follows:

Days from drug ingestion to hair collection.

Peak (1): [15 mm (Specimen 1, 15 mm; Specimen 2, 15 mm)] + [5 mm])/[0.4 mm/day] = 50 days (approximately 2 months).

Peak (2): [80 mm (Specimen 1, 82 mm; Specimen 2, 77 mm)] + [5 mm])/[0.4 mm/day] = 213 days (approximately 7 months).

#### Amount of intake

Focusing on the two ZP peaks, the total amount of ZP in the nine sections surrounding peak (1) (74 pg and 68 pg, corresponding to 1200 pg/mg and 1100 pg/mg, respectively) was 4 to 5 times higher than in the nine sections surrounding peak (2) (15 pg and 16 pg, corresponding to 240 pg/mg and 250 pg/mg, respectively). The amount of ZP detected around peak (2), which corresponds to the drug intake about 7 months ago, was lower than that detected under the condition of a single dose, suggesting that ZP may have leached out of the hair due to permanent hair coloring. Yegles. et al. [52] reported that benzodiazepine concentrations in hair decreased after bleaching (hydrogen peroxide treatment). Since the victim in this case underwent two permanent coloring treatments, both including bleaching, it is likely that the actual amount of ZP ingested was greater than one tablet. The authors have extensively examined the influences of hair dyeing and subsequent exposure to aqueous environments (e.g., hair washing) on drugs in hair, which will be discussed in the next section.

## Challenges in applying to various cases and future trends

### Influence of hair dyeing

The major weakness of hair testing is its susceptibility to the effects of hair dyeing. Many researchers have reported that bleaching or dyeing, particularly processes involving bleaching, decrease the amounts of drugs in hair [52, 64–68]. In DFSAs, victims often undergo hair dyeing at hair salons. When the victim has undergone dyeing after the incident, hair testing often have to be abandoned. However, drugs are not completely decomposed or removed by a single hair dyeing process. It is necessary to investigate how hair dyeing influences drug distribution and to determine the conditions under which hair testing could be performed. Thus, the authors investigated the influences of “semi-permanent coloring” and “permanent coloring” on drugs retention in hair [63].

The main differences between semi-permanent and permanent colorings involve two characteristics: the fixing position of the colorant and the degradation of melanin pigments. In semi-permanent coloring, the colorant adheres to the surface of the hair strand, but since melanin remains intact, it tends to be difficult to achieve the color change as imagined, particularly for black hair. In permanent coloring, on the other hand, the colorant penetrates the cortex where melanin has been decomposed, resulting in the desired color. For this reason, permanent coloring is generally preferred for dyeing black hair, which is common among Asians. However, hydrogen peroxide contained in permanent coloring agents, is known to reduce the concentration of drugs in hair.

**Fig. 7:**
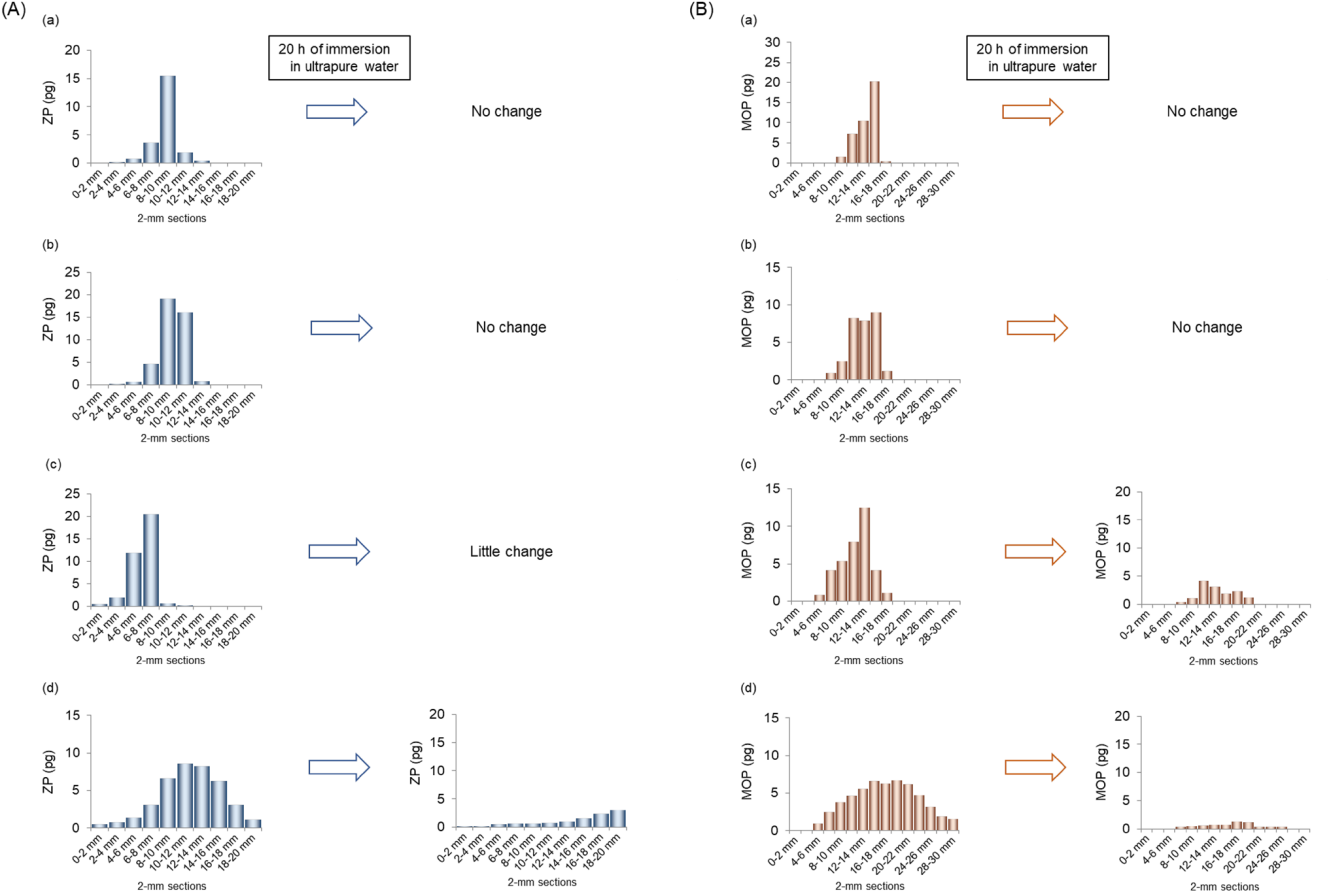
Amounts of **A** ZP and **B** MOP in each 2-mm segment along non-treated and dyed single strands collected from two subjects who took a single dose of ZP or MOP, before and after 20 h of immersion in ultrapure water [63]. (a), Non-treated; (b), Semi-permanent coloring; (c), Permanent coloring (once); (d), Permanent coloring (twice). *ZP* zolpidem, *MOP* methoxyphenamine

To evaluate the influence of hair dyeing on hair testing, the distribution shapes of drugs in individual hair strands after dyeing were investigated. Hair specimens were collected from subjects who had taken a single dose of ZP or MOP and then subjected to three different dyeing conditions: semi-permanent coloring, permanent coloring (once), and permanent coloring (twice) (Fig. 7). The results showed significant changes in the distribution shape under the permanent coloring (twice) condition, with a decrease in peak concentration and an extension of distribution width (Figs. 7A-d, B-d). In addition, the dyed hair specimens were immersed in ultrapure water for 20 h to simulate contact with aqueous environment (e.g., daily hair washing) after dyeing. Under the permanent coloring (twice) condition, most of both drugs in hair were leached out (ZP, 54–72%; MOP, 86–91%), flattening the concentration peaks making it impossible to estimate the date of intake. For MOP, although the majority (68–71%) was leached out of hair even under the permanent coloring (once), a slight concentration peak remained (Fig. 7B-c). These results indicate that both hair dyeing and subsequent exposure to aqueous environment significantly influence the amounts and distribution shapes of drugs in hair.

In a case of black hair, which is common among Asians, hair testing may be worthwhile if the victim had undergone hair dyeing only once, but if the hair has been dyed twice or more and has been exposed to water environment afterward, detecting drugs and reconstructing drug use history becomes nearly impossible. Since these findings and considerations are limited to black hair, further studies are necessary to establish baseline data for other hair colors such as brown, red, and blonde.

### Utilization for autopsy specimens

Hair testing is also expected to be valuable in autopsy cases. We recognize that autopsy specimens are rarely examined for detailed drug distribution in hair, especially the hair root. Nitta et al. [51] reported the drug distribution in the hair root in early stage (within 24 h) after MOP intake. Their study showed that drug incorporation begins approximately 30 min after intake, with peak incorporation from the hair bulb (region (1)) occurring at 3–10 h and in the upper dermis zone (region (2)) at 24 h after intake. These findings provide critical insights for forensic toxicology and the distribution of drugs in autopsy specimens.

In a past case, a victim was drugged with a hypnotic (TZ), tied up while unconscious, and then killed approximately 24 to 48 h after drug intake. Although the drug was detected in the victim's blood, its concentration was extremely low, falling below the therapeutic range. Blood analysis alone could not determine whether the drug had exerted a hypnotic effect on the victim. However, detailed analysis on drug distribution in the hair root can allow for the reconstruction of the victim’s most recent drug use history (frequency and the amount of intake). Thus, significant evidences related to incidents could be discussed using hair testing rather than urine or blood testing.

### Mass spectrometry imaging (MSI)

**Fig. 8:**
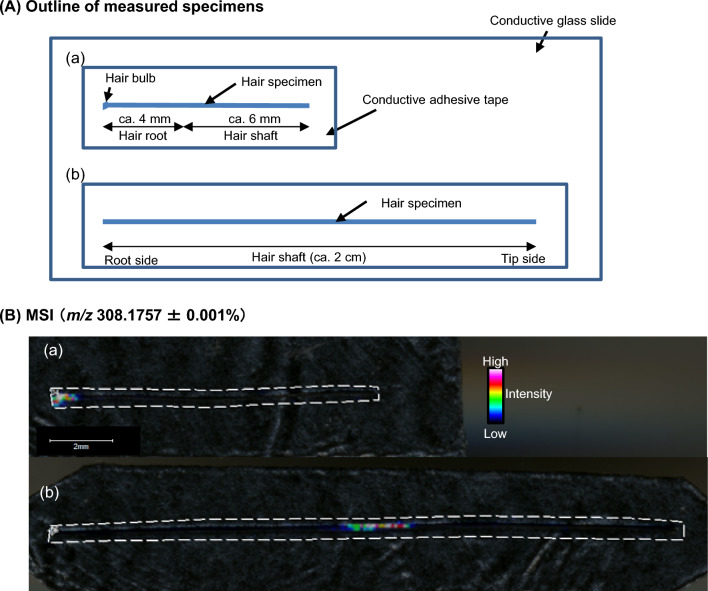
**A** Outline drawing of the measurement specimens and **B** MSI (*m/z* 308.1757 ± 0.0001%) of ZP on single hair strands collected from a subject after a single intake of 10 mg of ZP tartrate. The dotted fence lines represent the scanning regions [13]. (a), the 24-h specimen (plucked out); (b), the 35-day specimen (cut). *ZP* zolpidem

MSI by MALDI-MS has been reported as a technique for drug analysis in hair without chromatographic separation. The MSI technique was originally used to ionize drugs in biological tissues by direct laser irradiation, allowing for the visualization of drug distribution within a sample. MSI of drugs in hair was first reported by Miki et al. in 2011 for methamphetamine [69, 70], and has been applied to other abused drugs such as cocaine and ketamine, gaining worldwide recognition [71–75]. In 2015, the authors successfully imaged the distribution regions of ZP in hair following a single dose, which was the first application of MSI for hypnotics [10, 13]. Figure 8 shows the outline of measured specimens and the obtained MSI results at 24 h and 35 days after ZP intake [13]. At 24 h after intake, ZP was detected at a 0–1 mm distance from the root end, corresponding to the hair bulb. After 35 days, it was detected over a 2 mm width at about 1 cm from the root side. Referring to the distribution shape of ZP identified by LC–MS/MS (Fig. 3), it appears that MALDI-MS only detected the drug in regions of high concentration. In 2020, Kamata et al. utilized the high spatial resolution of MALDI-MS (5–10 µm) to successfully image not only longitudinal but also transversal sections (thickness of hair strand: 50–100 µm) of MOP in hair, demonstrating detailed visualization of drug distribution [75]. However, MALDI-MS is currently impractical for detecting benzodiazepines and orexin receptor antagonists in hair because of its much lower detection sensitivity compared to LC–MS/MS. To address this limitation, current research focuses on enhancing MALDI-MS performance and various pre-treatment techniques such as derivatization to improve detection sensitivity, promising further development in the future.

## Conclusion

Hair testing for hypnotics is gaining increasing attention in Japan, in part because of its effective application in DFSA and murder cases. Although the history is still short with many challenges, the demand for hair testing has exceeded the authors’ expectations, with many consultations received regarding serious crimes. This is one of the most difficult and intricate analyses in forensic toxicology, as it involves ultra-trace detection, requiring high-performance instruments and careful attention to external contamination. In addition, interpretation of results (estimation of the date and amount of intake) requires consideration of multiple factors, such as the chemical structures and physical properties of the detected drugs, their incorporation pathways, and the effects of hair dyeing. A thorough understanding of these fundamental findings is essential for accurate analysis. It is desirable that in the future hair testing becomes widely available in many laboratories, and for advancements in technology to enhance crime detection and deterrence. This review has summarized the findings on the concentration of hypnotics in hair and the incorporation pathways of drugs into hair, while providing as much reference data as possible for forensic applications. Additionally, this review has briefly outlined the currently recommended procedures for hair testing. With the continued development of new technologies and improvements in instrumental sensitivity, hair testing is expected to be refined into a method that can be performed quickly and accurately using smaller sample sizes.
